# The influence of entrepreneurial spirit on sports culture construction from the perspective of cognitive regulation

**DOI:** 10.3389/fpsyg.2022.980649

**Published:** 2022-09-15

**Authors:** Bo Pang, Hao Liu, Zhongqiang Liu

**Affiliations:** School of Physical Education, Southwest Medical University, Luzhou, Sichuan, China

**Keywords:** cognitive regulation, entrepreneurial spirit, sports culture, cultural construction, satisfaction survey

## Abstract

The purposes of this study are to extract the essence from the in-depth study of entrepreneurial spirit and the exploration of China’s sports culture construction, find out the beneficial parts in line with China’s sports culture construction, and put forward operational suggestions and countermeasures for dealing with the relationship between entrepreneurial spirit and sports culture to help China’s sports culture construction move toward a new level. This exploration discusses the influence of entrepreneurial spirit on sports culture from the perspective of cognitive regulation, and explores the importance of entrepreneurial spirit from the aspects of social psychology, system, economy, and ethics. The structure of sports culture is analyzed, and the role of entrepreneurial spirit in promoting the construction of sports culture is expounded. Moreover, a questionnaire survey is conducted to investigate the impact of entrepreneurial spirit on the construction of sports culture from the cognitive regulation perspective. The results show that most people agree with the development of the entrepreneurial spirit and positively impact the promotion of entrepreneurial spirit. Most people are not satisfied with the attention and level of sports culture construction. In China’s sports culture, the proportion of the entrepreneurial spirit is still tiny. Thereby, entrepreneurial spirit education must be vigorously promoted to enhance the integration of entrepreneurial spirit and sports culture. The entrepreneurial spirit needs to be introduced in the construction of sports culture. It is essential to do a good job in promoting entrepreneurial spirit, improving the supporting facilities related to sports culture and strengthening the correlation between sports culture and the entrepreneurial spirit. By discussing the influence of entrepreneurial spirit on sports culture, this exploration puts forward relevant opinions for developing sports culture. It is expected that they can have some reference values. This exploration provides new ideas for promoting the combination of sports culture construction and the dissemination of entrepreneurial spirit, which has crucial theoretical and practical significance.

## Introduction

### Research background

The term “entrepreneurial spirit” originated in French. It means to open up a new development way with the most active and creative behavior under uncertain conditions. It is to do something that others have not done, or to do something in a way that others have not taken. The most crucial characteristic of entrepreneurial spirit is creation. The essence of entrepreneurial spirit lies in the keen perception of opportunities that have not been realized before, which is an exploration of opportunities (Liu et al., 2019). Entrepreneurial spirit can be both the personality traits of entrepreneurs and the action characteristics of enterprises (Nite and Edwards, 2021). It is a dynamic process, which creates a wealth of growth (Wickam et al., 2020). Entrepreneurial spirit refers to discovering opportunities and combining various resources under the existing resource environment to fully use and develop opportunities and generate value-added. It can be divided into five aspects: autonomy, innovation, risk-taking, initiative and active competition (Zhong et al., 2020). The key to entrepreneurial spirit is to be creative and systematic. Entrepreneurial spirit can be found in various companies, governments and non-profit organizations. Its essence is a kind of action, not a kind of personality. Anyone who dares to make decisions has the opportunity to become a person with entrepreneurial consciousness through continuous efforts (Ryan et al., 2021). The entrepreneurial spirit is a process of creating various values. It requires people to spend a lot of time and energy, bear the corresponding financial, psychological and social risks, and obtain economic and self-realization rewards (Pellegrini et al., 2020). It is a process that is not limited by any resources, can give full play to opportunities and bring value to enterprises (Escamilla-Fajardo et al., 2020). Psychologically, it is a kind of creativity of thinking and behavior. In a practical sense, it is to create new value for the market by exploring opportunities and organizing resources to establish new companies.

The 16th National Congress of the Communist Party of China put forward that “the socialist society is a conscious social system established after the socialist culture and social development have reached a certain historical stage.” In the report of the 17th National Congress of the Communist Party of China, the great significance of strengthening cultural construction was particularly emphasized. It was based on the country’s cultural prosperity and development and aimed at creating a positive social atmosphere (Zheng and Liu, 2020). Hence, China’s excellent traditional culture should be vigorously developed to build a harmonious, healthy and advanced socialist country. China’s socialist civilization is a conscious unity of socialist culture developed gradually in the process of building a harmonious society in the early stage of socialist construction. With the integration of culture, industry and other fields, culture’s diversified development is more in-depth, and the demand for culture is increasingly prominent. The sports culture is a critical component and carrier of China on the international stage, and its construction degree plays a vital role in the content of national cultural construction.

Taking into account the above research, the objectives of the study are to clarify the connotation of cognitive regulation mechanism and the necessity of entrepreneurial spirit cultivation, and analyze the structure of sports culture. It is clear that the entrepreneurial spirit can promote the construction of sports culture. The questionnaire survey is conducted to investigate the impact of entrepreneurial spirit on the sports culture construction from the perspective of cognitive regulation. Operable suggestions and countermeasures to deal with the relationship between entrepreneurial spirit and sports culture are put forward. Integrating entrepreneurial spirit into the construction of sports culture is also a research innovation.

### Cognitive regulation mechanism

Cognitive regulation is a kind of psychotherapy. Cognitive psychology was a psychological trend in the West in the mid-1950s. It is about human higher thinking activities, including attention, perception, representation, memory, thinking, language and other cognitive activities. From the perspective of information processing, it is the development trend of cognitive psychology today. It can be said that it is equivalent to information processing psychology (Stroe et al., 2020). People are regarded as a processing system or a processing process, which means the transformation, simplification, processing, storage and use of perceptual input. Perception can be divided into several different periods according to this theory. Each period performs some specific processing on the obtained information, and the resulting response is the result of these processes and operations (Bryan et al., 2019). To some extent, the different components of the information processing system are connected to each other. Cognitive psychologists tend to divide information processing into several steps, thus causing people to understand the information flow in the body (Wardana et al., 2020). They usually use the time learning method. First, the time required for the process must be measured before the nature of the process can be determined. If a person is watching the letter E dropped on a fluorescent screen, this person will not see anything in a short millisecond, which means perception is not instantaneous. If the projection lasts longer, for example, five milliseconds, the person can see something unknown. It shows that perception has emerged, but has not yet formed. If the projection time is long enough for this person to distinguish that the letter is not O or Q, but whether the letter is E, F and K cannot be distinguished, the person has a certain resolution ability. From this point, it can be judged how long it takes to fully distinguish, partially distinguish or just see some objects (Alaassar et al., 2020).

By using the perspective of “cognitive regulation” of social cognition, it can be analyzed from the two aspects of “organizational environment” and “work situation”. It means that under the conditions of “organizational environment” and “work situation”, the “self” behavior of “employees” refers to the “self-regulation” of China’s “self-consciousness”. “Cognitive regulation” is to guide individuals’ actions through cognitive regulation of individuals (Lim et al., 2020).

### The necessity of cultivating entrepreneurial spirit

(1) Humanistic environment

From the humanism perspective, entrepreneurial spirit is a kind of spiritual characteristic of people with a special humanistic atmosphere. It is related to world outlook, values, philosophy of life, ethics, psychological needs and society’s mainstream culture. China has a fine tradition and rich cultural heritage. However, due to various factors, there is strong inertia in the inheritance of many excellent cultural factors, which hinders the development of entrepreneurial spirit in the modern market economy (Ratten and Jones, 2020). For example, the “golden mean” only adheres to its original intention and does not absorb its new content, which will inevitably exclude contradictions, innovation, enterprising, competition and adventure. The strong sense of “official rank standard” produced by the Confucian tradition has hindered the development of the entrepreneurial spirit, thus hindering the alienation of the function and action of the entrepreneurial spirit. Confucianism’s “not paying attention to business” thought makes the value of entrepreneurial spirit lack support. Misunderstanding and intolerance of entrepreneurs are the biggest obstacles for individuals to reject entrepreneurial spirit (Chang and Chen, 2020).

(2) System environment

The entrepreneur system is a series of institutional arrangements in the aspects of entrepreneur marketization, professionalization, incentive and restriction, assessment and training. It is an indispensable part of the modern company system. Meanwhile, it provides a solid organizational and talent foundation for the development of enterprises, and encourages and accelerates the development of entrepreneurs (Gordon and Reith, 2019). The socialist market economy has been initially established and the enterprise system has become the foundation of Chinese enterprises. However, problems such as the lack of separation between government and enterprises and the lack of vitality in state-owned enterprises are still very prominent due to the lag of the state-owned enterprise reform. They result in the imperfection of the sports system and the absence of sports culture, which is the direct reason for the lack of entrepreneurial spirit. It is also a fundamental institutional constraint for the healthy growth of Chinese entrepreneurs and the healthy development of sports culture (Haddoud et al., 2022).

(3) Economy and ethics

Economic ethics, as mentioned earlier, is the sum of values, ethics and ethical spirit. They form in social life, restrict and adjust human economic behavior and the links between them (Sacco et al., 2018). Professor Luoshifan, a Swiss Economics ethicist, believed that corporate ethics is the ultimate goal of entrepreneurs for the development of human society. It can improve the living conditions of the company, further develop the company, and provide the most fundamental guarantee for company’s sustainable development. It is also believed that China has become an important power source in Asia, and China is also a vital driving force of economic and moral issues. China’s development will be a new problem. In addition to the rapid speed of development, sustainable development should not be neglected. The guiding role of economic ethics in enterprise management should be noted (Lee et al., 2021).

### Structure analysis of sports culture

**FIGURE 1:**
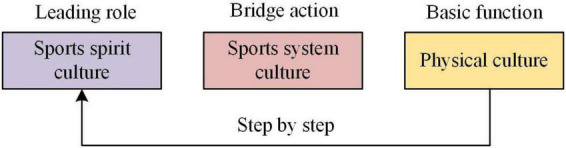
Division of sports culture levels.

The structure of sports culture determines the characteristics of sports culture. It is a cultural form with extensive connotation and an open system (Xu et al., 2021). From the culturology perspective, sports culture can be divided into three levels (Figure 1): the surface physical culture layer (mainly including campus sports facilities, sports equipment, sports venues, sports sculptures, sports clothing and sports goods); middle-level behavior system culture level (school sports tradition, standardized sports teaching, extracurricular sports activities, extracurricular sports culture construction, rules and regulations for training and competition, various school sports organizations); the inner spiritual culture layer (sports concept, sports thinking mode, sports spirit and values, and sports knowledge). These three levels have a progressive relationship (Rathwell and Young, 2018). Among them, the sports spiritual culture layer is the core and soul of sports culture, which occupies the leading position in sports culture. The sports system culture layer is the middle layer connecting the spiritual and material cultural layers, and plays a bridge role to the other two layers. The sports physical culture layer is the foundation and material guarantee of sports culture construction (McDougall et al., 2020). These three levels are the subsystem of the sports culture system. They promote each other, contact each other and develop together. The formation of good sports culture requires the construction of these three levels under a certain macro environment. Figure 1 displays the division of sports culture levels.

### The role of entrepreneurial spirit in promoting the construction of sports culture

(1) Entrepreneurial spirit guides sports culture construction

The entrepreneurial spirit has promoted the construction of sports culture in China. The idea of “dare to take on responsibilities, assume responsibilities and serve the society” advocated by the entrepreneurial spirit is a kind of firm confidence in life, a kind of enterprising sports spirit and a kind of cultivation of the entrepreneurial spirit. It will enable people to form a positive and enterprising personality and the spirit of struggling in the fierce competition. In learning, it can cultivate people to overcome various difficulties in life and work, face various challenges with a positive attitude, and benefit from them for life (Hoang et al., 2020). The entrepreneurial spirit emphasizes creation. The enterprising spirit of sports culture construction embodied in entrepreneurial spirit has been widely spread in this period, and has formed a code of conduct and values to a certain extent (Ratten, 2020). Physical education can encourage people to establish correct moral concepts and values, promote people to establish fair and just moral concepts, and reduce people’s moral corruption in competition. It can make people explore their potential, enhance their competitiveness, and actively participate in social competition (Ullah et al., 2021). Entrepreneurial spirit advocates tolerance, understanding of different cultures and countries, and the idea of harmony. It helps to cultivate people’s understanding of the world, thus promoting social stability and coordination (Elnadi and Gheith, 2021).

(2) Entrepreneurial spirit can strengthen the educational function of sports culture construction

Physical education should attract the general public to participate in and strengthen their comprehensive training in sports culture construction concepts, habits, health and comprehensive quality. In physical education, the knowledge of sports culture construction should be popularized. Besides, people should turn scientific health education concepts and practical activities into their own thinking and intelligence to correctly understand the sports culture construction and improve their values on it (Raggiotto et al., 2020). The entrepreneurial spirit has irreplaceable significance for the morality and social norms of the younger generation (Bonesso et al., 2018). Therefore, the deeper the understanding of the sports cultural characteristics is, the more the sports can meet the global youth and become a link among all ethnic groups (Chavoushi et al., 2021). Therefore, the entrepreneurial spirit can strengthen the cultivation of people’s sports culture.

(3) Entrepreneurial spirit promotes the deepening and opening of sports culture construction

The modern world is open and moving forward. The entrepreneurial spirit is the forming process of an open and dynamic sports culture system, and it is a cross-cultural, cross-ethnic and cross-national spiritual ideology (Van der Veken et al., 2020). Its “generic” generation and communication mode put forward a more in-depth and open development concept for China’s sports culture construction. It also encourages the broad masses to go out of school and go to the world more firmly, and actively participate in the worldwide sports culture construction to learn from advanced experience, thus better promoting the healthy and steady development of China’s sports culture while continuously expanding the opening-up.

## Materials and methods

The influence of entrepreneurial spirit on the establishment of sports culture is reasonably understood and investigated to deeply understand the situation of the integration of entrepreneurial spirit and sports culture.

(1) Survey objects

The survey objects are sports practitioners, athletes, college students and the general public in XX Province. Overall, 200 people are selected by random sampling.

(2) Survey method

The survey is conducted by questionnaire. In order to get accurate information about the current combination of entrepreneurial spirit and sports culture construction, a questionnaire “Questionnaire on the Current Situation of the Combination of Entrepreneurial Spirit and Sports Culture Construction” is designed for college students, teachers and experts. Experts and professors of sports culture construction in some colleges are visited. Experts’ opinions and suggestions for the test indicators in the questionnaire are solicited many times, and effective supplements and modifications are made accordingly. Experts are invited to analyze and evaluate the questionnaire to ensure its validity. The average validity score of the questionnaire is 8.70 (full score: 10). The questionnaires are distributed after being unanimously approved by experts. The test-retest reliability of this questionnaire is *r* = 0.81, which proves that it has available reliability and validity.

The questionnaire survey is mainly conducted online and on-site. Overall, 200 questionnaires were collected, 195 of which were valid. The total effective rate was 97.55%.

## Results and discussion

The starting point and destination of entrepreneurial spirit is social development. In order to understand the current situation of the combination of entrepreneurial spirit and sports culture construction, and to have a rational understanding of the role of entrepreneurial spirit in sports culture construction, a survey of the current situation is carried out. The following survey results are obtained through the statistics and analysis of the questionnaire data:

### Analysis on the survey results of entrepreneurial spirit

**FIGURE 2:**
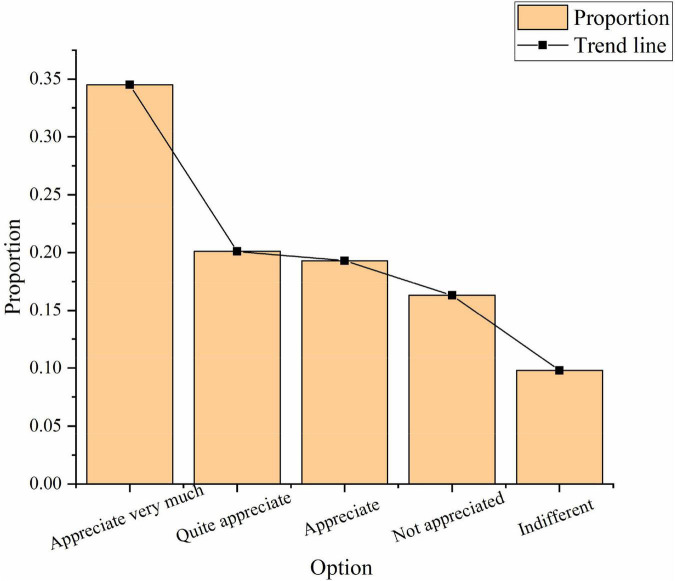
Recognition of entrepreneurial spirit.

**FIGURE 3:**
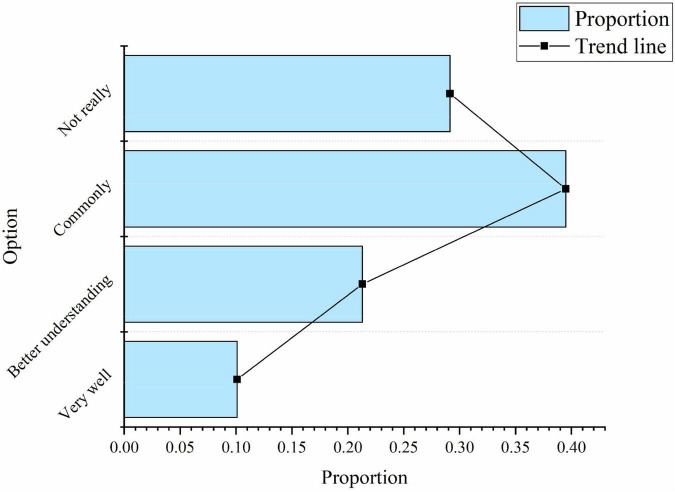
Understanding of entrepreneurial spirit.

**FIGURE 4:**
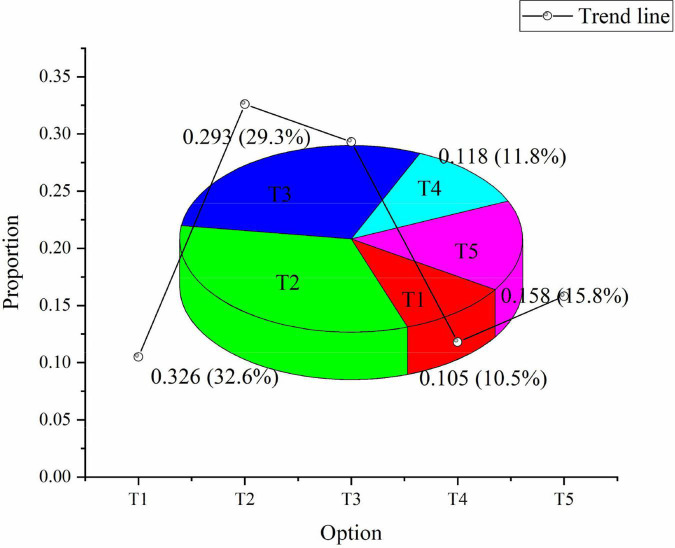
Social role of entrepreneurial spirit. T1: it can promote the construction of spiritual civilization. T2: it will improve people’s quality. T3: it can promote social harmony. T4: it can promote environmental protection. T5: others.

**FIGURE 5:**
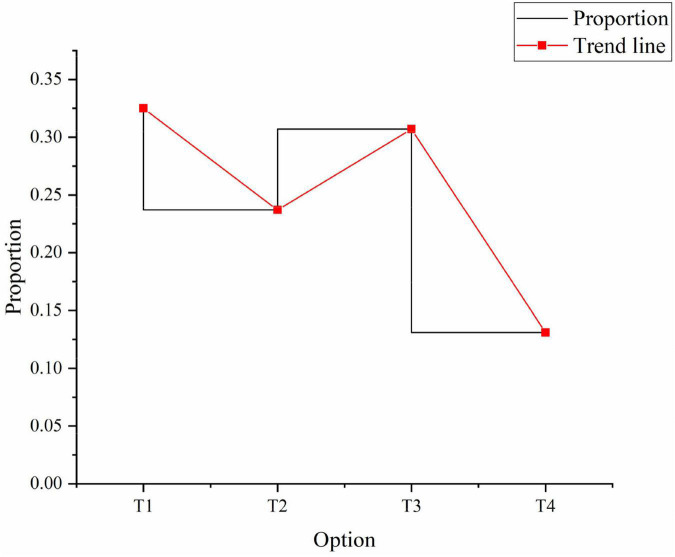
The impact of entrepreneurial spirit on people. T1: it can improve personal accomplishment. T2: it can promote the construction of sports culture. T3: it can improve interest in sports culture construction. T4: others.

The public’s present understanding of entrepreneurial spirit has been studied. In recognition of entrepreneurial spirit, 34.5.% of the respondents highly affirm this, 20.1% agree, 19.3% disagree, and 9.8% do not care, as shown in Figure 2:

In terms of the level of understanding of entrepreneurial spirit, 10.1% of the people think they know it very well, 21.3% hold that they have a better understanding, 39.5% believe that they have a general understanding, and 29.1% hold that they do not really know it (Figure 3):

As for the social role of entrepreneurial spirit, 10.5% of the people think it can promote the construction of spiritual civilization. 32.6% think it will improve people’s quality. 29.3% hold that it can promote social harmony. 11.8% think it can promote environmental protection. 15.8% choose others (Figure 4):

As for the impact of entrepreneurial spirit on people themselves, 32.5% of people think it can improve their personal quality. 23.7% hold that it can promote the sports culture construction. 30.7% say that it can improve their interest in the sports culture construction, and 13.1% choose others, as shown in Figure 5:

The above data show that the vast majority of people agree with the development of the entrepreneurial spirit, and they have positively impacted the promotion of entrepreneurial spirit. They believe that entrepreneurial spirit positively impacts the development of the whole country. However, there are still differences in how to understand entrepreneurial spirit.

### Analysis of the investigation results of sports culture construction

**FIGURE 6:**
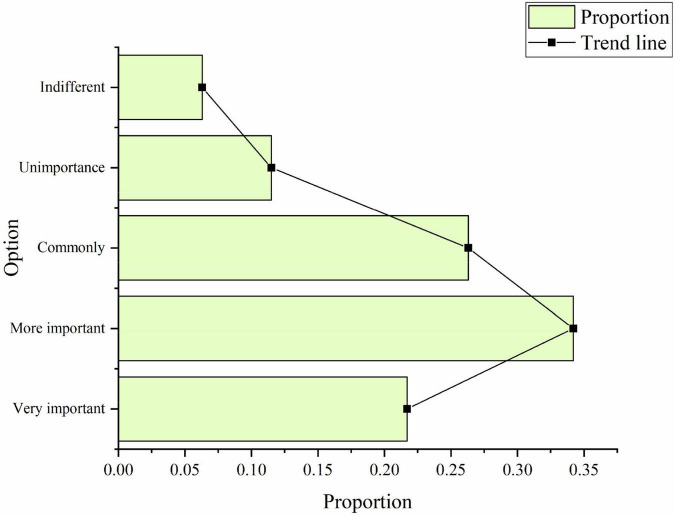
Investigation on the importance of sports culture construction.

**FIGURE 7:**
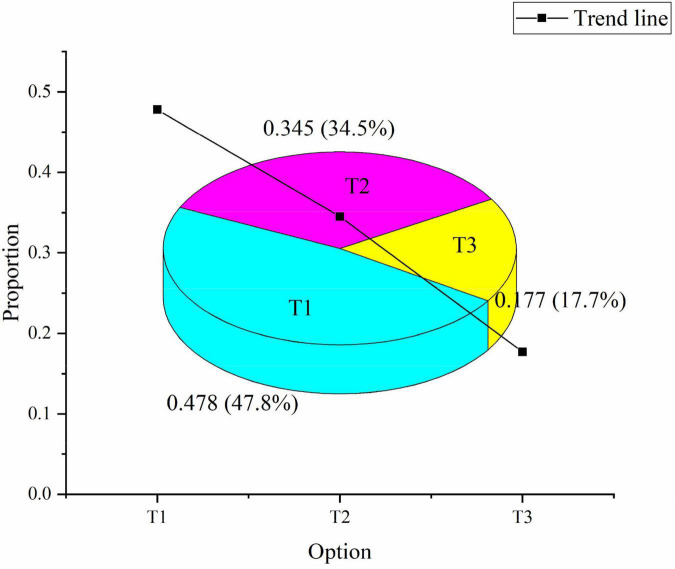
Investigation of the necessity of sports culture construction. T1: the construction of sports culture is an indispensable part. T2: unnecessary. T3: it doesn’t matter.

**FIGURE 8:**
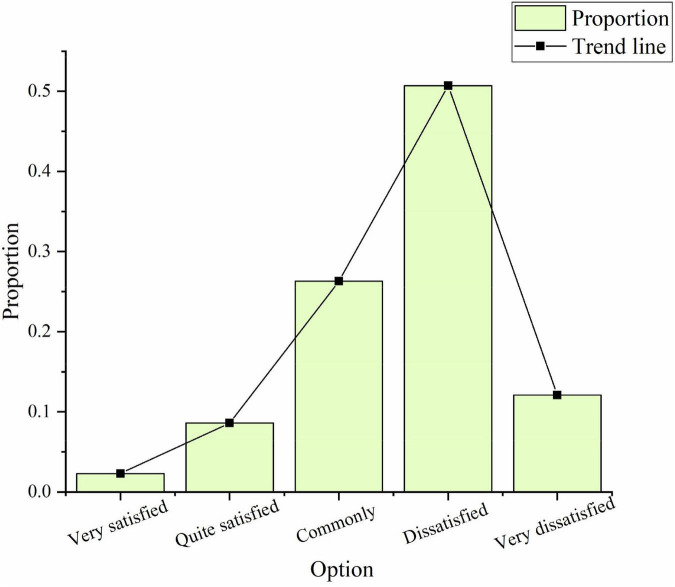
Investigation of the necessity of sports culture construction.

Understanding and participating in sports culture construction is a key link affecting the development of China’s sports cause. Only by deeply understanding the public’s understanding of sports culture construction can people better analyze the integration of entrepreneurial spirit and sports culture construction. In the questionnaire on the importance of sports culture construction, the proportions of people choosing “very important,” “more important,” “commonly,” “unimportant,” and “indifferent” are 21.7%, 34.2%, 26.3%, 11.5% and 6.3% (Figure 6):

This point reveals that the general public has widely recognized the significance of sports culture. Therefore, more attention has been paid to sports culture construction. 47.8% of the people put forward higher requirements for the development of sports culture, 34.5% of the people think it is unnecessary, and 17.7% of the people think it doesn’t matter, as shown in Figure 7:

In the survey on the actual situation of sports culture construction, only 2.3% of the people show great satisfaction, 8.6% show relatively satisfaction, 26.3% show general satisfaction, 50.7% feel dissatisfied, and 12.1% feel very dissatisfied, as shown in Figure 8:

The above data prove that most people are not satisfied with the importance and construction of sports culture.

### Analysis on the influence of entrepreneurial spirit on sports culture construction from the perspective of cognitive regulation

**FIGURE 9:**
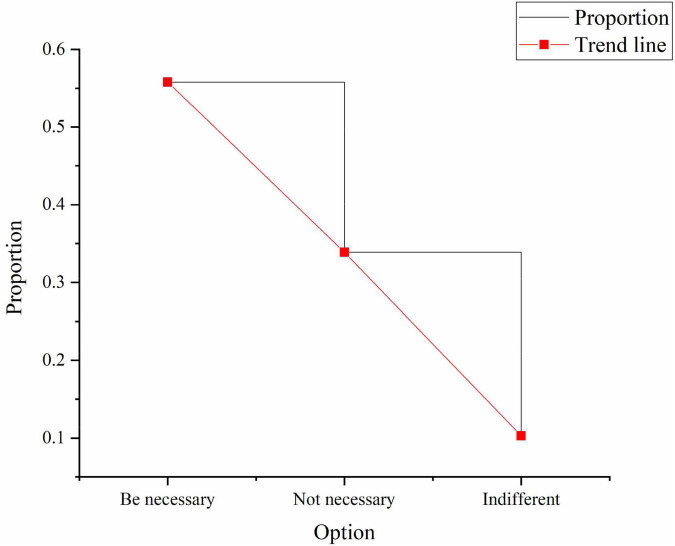
Investigation of the integration of entrepreneurial spirit into the construction of sports culture.

In the survey results of “recognition of the integration of entrepreneurial spirit into the construction of sports culture”, 55.8% choose it as necessary, 33.9% choose it as unnecessary, and 10.3% think it doesn’t matter, as shown in Figure 9:

Figure 9 suggests that most practitioners and managers believe that in the process of entrepreneurship, they will agree more with the concept of entrepreneurial spirit and are willing to explore the role of entrepreneurial spirit education.

**FIGURE 10:**
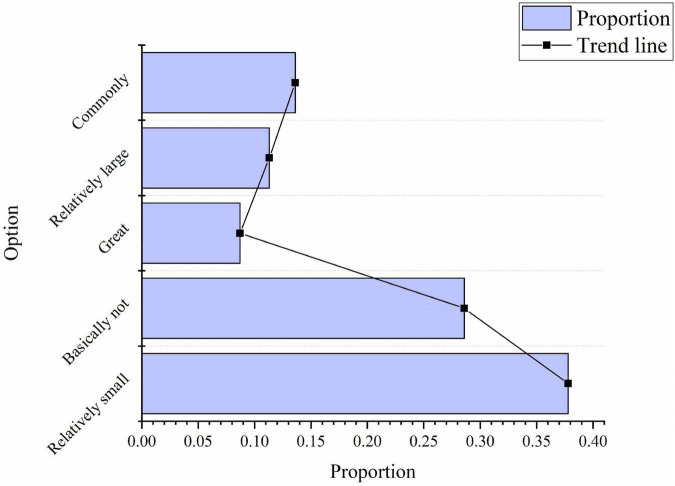
Investigation of the proportion of entrepreneurial spirit in the construction of sports culture.

**FIGURE 11:**
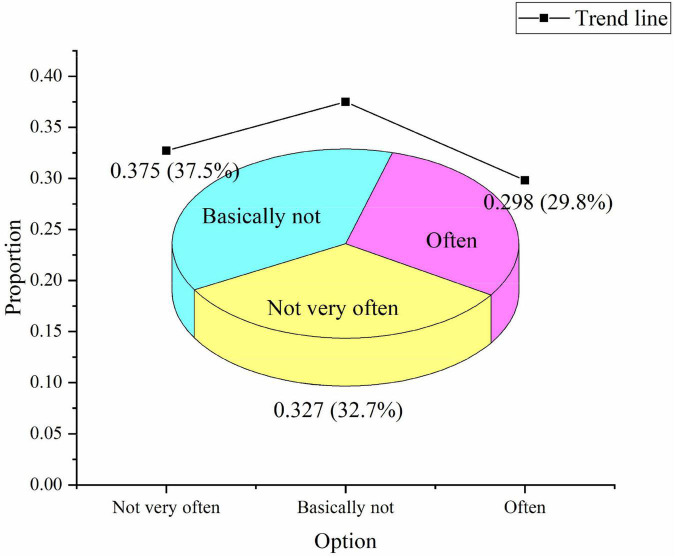
Investigation of similar activities such as sports culture lectures.

In the survey on the proportion of entrepreneurial spirit in the current sports culture construction, 37.8% of the people choose “relatively small,” 28.6% of them choose “basically none,” 8.7% choose “great,” 11.3% choose “relatively large,” and 13.6% choose “commonly,” as shown in Figure 10:

In terms of whether the society organizes sports lectures and other similar activities, 32.7% think it is less frequent, 37.5% think it is basically not, and 29.8% think it is frequent, as shown in Figure 11:

The above data suggest that the current practice effect in promoting entrepreneurial spirit is not very good, which is far from people’s needs. In the development of sports culture, the proportion of the entrepreneurial spirit is still tiny. Most people feel that entrepreneurial spirit should be vigorously promoted. It is essential to use all kinds of human and material resources, organize sports culture publicity with relevant sports institutions and professionals, and encourage more people to organize similar activities.

## Discussion

Through the understanding and adjustment of entrepreneurial spirit activities, this exploration reveals the important significance of entrepreneurial spirit in sports culture construction. It provides useful enlightenment on how to introduce entrepreneurial spirit into China’s sports culture and sports, and how to promote the development of China’s sports undertakings. The establishment of sports culture in China is discussed, and some suggestions are put forward for the development. It has significant practical value for developing the country’s cultural soft power and enhancing its overall strength, national cohesion and innovation awareness.

This exploration discusses the influence of entrepreneurial spirit on the sports culture establishment from the perspective of cognitive regulation. Improvement should be made mainly from the following aspects. In the aspect of conducting entrepreneurial spirit education, it is essential to establish entrepreneurial spirit education institutions and strengthen theoretical research. Moreover, the entrepreneurial spirit should be integrated into college teaching and social practice. The teaching staff of sports culture education and public welfare sports clubs should be established. Social entrepreneurial spirit exchange activities and various sports and competitive activities in society and colleges need to be actively conducted. In the construction of the cultural environment, it is essential to do a good job in the publicity of entrepreneurial spirit, improve the construction of sports-related facilities, increase the content of entrepreneurial spirit, and further integrate entrepreneurial spirit into the sports culture construction.

## Conclusion

From the perspective of cognitive adjustment, this exploration discusses the influence of entrepreneurial spirit on the sports culture establishment. (1) The necessity of entrepreneurial spirit education is discussed from aspects of cognition and regulation mechanism, humanities, system, economy and ethics. The critical significance of entrepreneurial spirit education in promoting the sports culture establishment in China is illustrated by analyzing the organizational structure of entrepreneurial spirit education. (2) The vast majority of the people agree with the entrepreneurial spirit. They all have a positive impact on the promotion of entrepreneurial spirit. They believe that entrepreneurial spirit positively impacts the whole country’s development, and they also highly agree with the entrepreneurial spirit improvement. Most people are not satisfied with the attention and level of sports culture construction. In China’s sports culture, the proportion of the entrepreneurial spirit is still tiny. (3) Entrepreneurial spirit education must be vigorously carried out to better integrate entrepreneurial spirit into the enterprises’ development. In sports culture construction, the entrepreneurial spirit should be introduced. It is essential to do a good job promoting the awareness of entrepreneurial spirit, strengthening the supporting facilities related to sports, and strengthening the relevant elements of entrepreneurial spirit.

However, there are still many problems worth discussing. As the research and construction of sports culture is a very long-term, professional and complex systematic work, the relevant research on the construction and development of sports culture worldwide has yielded numerous results. Due to the limitations of professional ability and knowledge level, a deeper discussion is not conducted here.

## Data availability statement

The raw data supporting the conclusions of this article will be made available by the authors, without undue reservation.

## Ethics statement

The studies involving human participants were reviewed and approved by Southwest Medical University Ethics Committee. The patients/participants provided their written informed consent to participate in this study. Written informed consent was obtained from the individual(s) for the publication of any potentially identifiable images or data included in this article.

## Author contributions

All authors listed have made a substantial, direct, and intellectual contribution to the work, and approved it for publication.

## References

[B1] Alaassar A. Mention A. L. Aas T. H. (2020). Exploring how social interactions influence regulators and innovators: The case of regulatory sandboxes. *Technol. Forecast. Soc. Change* 160:120257. 10.1016/j.techfore.2020.120257

[B2] Bonesso S. Gerli F. Pizzi C. Cortellazzo L. (2018). Students’ entrepreneurial intentions: The role of prior learning experiences and emotional, social, and cognitive competencies. *J. Small Bus. Manage.* 56 215–242. 10.1111/jsbm.12399

[B3] Bryan C. O’Shea D. MacIntyre T. (2019). Stressing the relevance of resilience: A systematic review of resilience across the domains of sport and work. *Int. Rev. Sport Exerc. Psychol.* 12 70–111. 10.1080/1750984x.2017.1381140

[B4] Chang Y. Y. Chen M. H. (2020). Creative entrepreneurs’ creativity, opportunity recognition, and career success: Is resource availability a double-edged sword? *Eur. Manage. J.* 38 750–762. 10.1016/j.emj.2020.03.004

[B5] Chavoushi Z. H. Zali M. R. Valliere D. Faghih N. Hejazi R. Dehkordi A. M. (2021). Entrepreneurial alertness: A systematic literature review. *J. Small Bus. Entrepreneur.* 33 123–152. 10.1007/978-3-642-31098-0_2

[B6] Elnadi M. Gheith M. H. (2021). Entrepreneurial ecosystem, entrepreneurial self-efficacy, and entrepreneurial intention in higher education: Evidence from Saudi Arabia. *Int. J. Manage. Educ.* 19:100458. 10.1016/j.ijme.2021.100458

[B7] Escamilla-Fajardo P. Núñez-Pomar J. M. Gómez-Tafalla A. M. (2020). Exploring environmental and entrepreneurial antecedents of social performance in Spanish sports clubs: A symmetric and asymmetric approach. *Sustainability* 12:4234. 10.3390/su12104234

[B8] Gordon R. Reith G. (2019). Gambling as social practice: A complementary approach for reducing harm? *Harm Reduct. J.* 16:64. 10.1186/s12954-019-0342-2 31805952 PMC6896290

[B9] Haddoud M. Y. Onjewu A. K. E. Nowinski W. Alammari K. (2022). Assessing the role of entrepreneurship education in regulating emotions and fostering implementation intention: Evidence from Nigerian universities. *Stud. High. Educ.* 47 450–468. 10.1080/03075079.2020.1758652

[B10] Hoang G. Wilson-Evered E. Lockstone-Binney L. (2020). Leaders influencing innovation: A qualitative study exploring the role of leadership and organizational climate in Vietnamese tourism SMEs. *Employee Relations* 43 416–437. 10.1108/er-07-2019-0279

[B11] Lee J. S. Kwak D. H. Bagozzi R. P. (2021). Cultural cognition and endorser scandal: Impact of consumer information processing mode on moral judgment in the endorsement context. *J. Bus. Res.* 132 906–917. 10.1016/j.jbusres.2020.11.029

[B12] Lim J. S. Choe M. J. Zhang J. Noh G. Y. (2020). The role of wishful identification, emotional engagement, and parasocial relationships in repeated viewing of live-streaming games: A social cognitive theory perspective. *Comp. Hum. Behav.* 108:106327. 10.1016/j.chb.2020.106327

[B13] Liu Q. Cheng Z. Chen M. (2019). Effects of environmental education on environmental ethics and literacy based on virtual reality technology. *Electron. Library* 37 860–877. 10.1108/el-12-2018-0250

[B14] McDougall M. Ronkainen N. Richardson D. Littlewood M. Nesti M. (2020). Three team and organisational culture myths and their consequences for sport psychology research and practice. *Int. Rev. Sport Exerc. Psychol.* 13 147–162. 10.1080/1750984x.2019.1638433

[B15] Nite C. Edwards J. (2021). From isomorphism to institutional work: Advancing institutional theory in sport management research. *Sport Manage. Rev.* 24 815–838. 10.1080/14413523.2021.1896845

[B16] Pellegrini M. M. Rialti R. Marzi G. Caputo A. (2020). Sport entrepreneurship: A synthesis of existing literature and future perspectives. *Int. Entrepreneur. Manage. J.* 16 795–826. 10.1007/s11365-020-00650-5

[B17] Raggiotto F. Scarpi D. Moretti A. (2020). Advertising on the edge: Appeal effectiveness when advertising in extreme sports. *Int. J. Advertising* 39 655–678. 10.1080/02650487.2019.1653009

[B18] Rathwell S. Young B. W. (2018). Coaches’ perspectives on personal and psychosocial development in university sport. *Int. Sport Coach. J.* 5 1–13. 10.1123/iscj.2017-0018

[B19] Ratten V. (2020). Sport technology: A commentary. *J. f High Technol. Manage. Res.* 31:100383. 10.1016/j.hitech.2020.100383

[B20] Ratten V. Jones P. (2020). New challenges in sport entrepreneurship for value creation. *Int. Entrepreneur. Manage. J.* 16 961–980. 10.1007/s11365-020-00664-z

[B21] Ryan R. M. Deci E. L. Vansteenkiste M. Soenens B. (2021). Building a science of motivated persons: Self-determination theory’s empirical approach to human experience and the regulation of behavior. *Motivat. Sci.* 7 97–110. 10.1037/mot0000194

[B22] Sacco P. L. Ferilli G. Tavano Blessi G. (2018). From culture 1.0 to culture 3.0: Three socio-technical regimes of social and economic value creation through culture, and their impact on European Cohesion Policies. *Sustainability* 10:3923. 10.3390/su10113923

[B23] Stroe S. Sirén C. Shepherd D. Wincent J. (2020). The dualistic regulatory effect of passion on the relationship between fear of failure and negative affect: Insights from facial expression analysis. *J. Bus. Ventur.* 35:105948. 10.1016/j.jbusvent.2019.105948

[B24] Ullah F. Wu Y. Mehmood K. Jabeen F. Iftikhar Y. Acevedo-Duque Á (2021). Impact of spectators’ perceptions of corporate social responsibility on regional attachment in sports: Three-wave indirect effects of spectators’ pride and team identification. *Sustainability* 13:597. 10.3390/su13020597

[B25] Van der Veken K. Lauwerier E. Willems S. J. (2020). How community sport programs may improve the health of vulnerable population groups: A program theory. *Int. J. Equity Health* 19:74. 10.1186/s12939-020-01177-5 32448226 PMC7245920

[B26] Wardana L. W. Narmaditya B. S. Wibowo A. Mahendra A. M. Wibowo N. A. Harwida G. (2020). The impact of entrepreneurship education and students’ entrepreneurial mindset: The mediating role of attitude and self-efficacy. *Heliyon* 6:e04922. 10.1016/j.heliyon.2020.e04922 32995616 PMC7502346

[B27] Wickam M. J. Finley L. R. Saeger K. (2020). Assessing alignment of Entrepreneurial Spirit to job descriptions seeking business administration or management undergraduates. *J. Educ. Bus*. 95, 527–533. 10.1080/08832323.2020.1715332

[B28] Xu D. Zheng Y. Jia Y. (2021). The bibliometric analysis of the sustainable influence of physical education for university students. *Front. Psychol.* 12:592276. 10.3389/fpsyg.2021.592276 33746825 PMC7971110

[B29] Zheng Y. Liu S. (2020). Bibliometric analysis for talent identification by the subject–author–citation three-dimensional evaluation model in the discipline of physical education. *Library Hi Tech* 40 62–79. 10.1108/lht-12-2019-0248

[B30] Zhong H. Yan R. Li S. Chen M. (2020). The psychological expectation of new project income under the influence of the entrepreneur’s sentiment from the perspective of information asymmetry. *Front. Psychol.* 11:1416. 10.3389/fpsyg.2020.01416 32774311 PMC7381333

